# Some Curious Fashions, Old and New

**Published:** 1920-12-18

**Authors:** 


### PSYCHO-ANALYSIS.

### Some Curious Fashions, Old and New.

There is a certain type of person who, in all ages, persists in crying out for a new thing, and, when it has been vouchsafed to him, calls upon all to unite with him in praising it. It matters not whether it be worthy of praise or not: it is new and strange, and that is all-sufficing. Nowhere is this more common than in the realms of medicine, and possibly in no region thereof so frequently to be observed as in the matter of mental disorders. Of course, it is difficult for any promulgators of mental "cures" to vie with the deeds of healers of cancer or of tuberculosis; but these are not so "occult" as mental afflictions, and consequently they cannot be surrounded with such an atmosphere of mystery. Nor are they so liable to such fluctuations, though even in those disorders there are remissions and spontaneous cures sufficient to make the reputations of many "healers" and, let it be said with bated breath, of not a few medical men.

### Past History.

Even a perfunctory study of the history of medicine will convince anyone that, no matter how absurd the hypothesis may be, it will always find ardent supporters who, if they have the power, will make it very uncomfortable for those who do not agree with them. Demoniacal possession as a cause of insanity has still its advocates, and exorcism is practised in as good faith as ever. Miracle-working images and shrines are fervently believed in. Variegated electricity is extolled by retired generals who have had their mentality sapped by years of Army routine. Mesmer, Cagliostro, and Paracelsus are dead, but Brown, Jones, and Robinson rule in their stead.* "Mental healing" has been the prerogative of no one caste or class. For the most part the possession of any scientific knowledge has been rather a drawback. It hampers the imagination, which, as everyone knows, is the most important adjunct. Given a vivid imagination, a smattering of metaphysical verbiage, a tendency towards mysticism, and a capacity for inventing neologisms, and you have the ingredients of systems which will revolutionise the thoughts—or the emotions—of the majority of the public. Especially potent will be the effect if the emotional appeal is made by way of some theological system or if there is sexual suggestiveness—in many instances the wily ones of the earth combine a little of both.

### The Medical Profession.

The medical profession is often spoken of as a body which is so intensely conservative that it fails to be influenced by anything novel, and that it acquiesces in a new doctrine only after years of opposition. In this connection the name of Harvey is usually invoked, and no one will deny that there has at times been an unwarrantable delay in incorporating new knowledge into the medical canon. But, on the other hand, medical men are accused of an undue desire to experiment on the bodies of their patients, and this accusation is brought quite frequently by the same people who rail against them because of the non-adoption of the latest crank or fad! On the whole, the public, weighing one thing against another, may congratulate itself that medical opinion is not swayed by every breath of theory.

### A Modern Tendency.

Yet, despite the conservatism, only too often some ill-digested hypothesis runs rife through the medical body politic! Witness the latest importation, psycho-analysis—though even as the words are written some other even more appalling disorder may be incubating. In this case the appeal was frankly through the channel of sex. Consideration of the amount of venereal disease, illegitimacy, exiguously dressed ladies upon many stages, the high prices paid for literature of the erotic variety, the hilarious reception accorded to smoke-room tales, makes it clear that the interest in matters sexual is fairly widespread. Then, too, the fact that in a decently organised society it is considered fitting to relegate pornographic literature to the top shelf and to refrain from bawdy talk until the ladies have departed demonstrates how we practise repression. Now, this repression of our desires into the subconsciousness—or into "the unconscious"—is a most reprehensible thing, and leads to all kinds of nervous ailments. The oracles have spoken, and it must be so. Obviously, then, it follows as the night the day that we must get rid of our repressions.

Incidentally, this reminds us of one of the chief articles of Oscar Wilde's creed anent expression to our desires. The process may be carried out in various ways. One is, apparently, to let things rip, and waive the consequences. The other is to submit to mental purgation or excavation by means of a modified confessional—heart to heart talks, with a running accompaniment of "free association." † Then there is a valuable adjunct in the shape of dream analysis, a modern form of oneiromancy. Each dream is supposed to represent an unfulfilled wish, sexual for the most part in its trend.

### The Climax.

It is difficult to know what to admire most, the fearsome ingenuity of the promulgators of these ideas or the guileless confidence of those who accept them. We have laughed at the arid and absurd disputations of the Middle Ages, but it seems as if our turn will yet come to be the laughing-stocks

* This Silas-Weggish sentence must be an upheaval of a sternly repressed complex consequent upon reading the works of certain modern poets. Also—apologies to Brown, etc., but the law of libel necessitates the invocation of their non-committal names!

† Lest this should give rise to misunderstanding, the word "free" is not used in the sense of indecent.

**Psycho-Analysis**—(*continued*).

for posterity. Even if we allow a kernel of truth to this codex it seems to be over-weighted with a vast mass of hypothesis and web-spinning—"but one half-pennyworth of bread to this intolerable deal of sack"! We can infer this from the intolerance with which Brown speaks of Jones' theories, a contempt only exceeded by that which Jones expresses in regard to poor Robinson! What is looked for in science is a seemly modesty in the face of age-long problems, and not dogmatic self-assertiveness.

If there is aught that is good in this inflated system that good will survive and be incorporated with other well-based knowledge—even as a residuum has been abstracted from the bombastic utterances of Paracelsus and others. In the meantime it can hardly be gainsaid that much harm is being done by the many practisers of this art. Many of them deny the effectiveness of suggestion, yet they have succeeded in inculcating nauseous and noxious doctrines into the minds of those who are singularly receptive of such ideas. Many an analysis is more valuable in demonstrating the—more or less—repressed ideas in the mind of the analyser rather than in that of the analysed. And—dangerous sign to anyone acquainted with the progress of such ideas—it has found acceptance with those who are on the look-out for fads and fancies.

